# Hyaluronic Acid in Rheumatology

**DOI:** 10.3390/pharmaceutics15092247

**Published:** 2023-08-30

**Authors:** Haiko Sprott, Christian Fleck

**Affiliations:** 1 Medical Faculty, University of Zurich (UZH), CH-8006 Zurich, Switzerland; 2 Arztpraxis Hottingen, CH-8032 Zurich, Switzerland; 3 Medical Faculty, University of Jena, D-07743 Jena, Germany; fleck-christian@t-online.de

**Keywords:** hyaluronic acid, rheumatology, osteoarthritis, rheumatoid arthritis, joint lubrication, cartilage, inflammation, therapeutic applications, combination therapies, patient outcomes

## Abstract

Hyaluronic acid (HA), also known as hyaluronan, is an anionic glycosaminoglycan widely distributed throughout various tissues of the human body. It stands out from other glycosaminoglycans as it lacks sulfation and can attain considerable size: the average human synovial HA molecule weighs about 7 million Dalton (Da), equivalent to roughly 20,000 disaccharide monomers; although some sources report a lower range of 3–4 million Da. In recent years, HA has garnered significant attention in the field of rheumatology due to its involvement in joint lubrication, cartilage maintenance, and modulation of inflammatory and/or immune responses. This review aims to provide a comprehensive overview of HA’s involvement in rheumatology, covering its physiology, pharmacology, therapeutic applications, and potential future directions for enhancing patient outcomes. Nevertheless, the use of HA therapy in rheumatology remains controversial with conflicting evidence regarding its efficacy and safety. In conclusion, HA represents a promising therapeutic option to improve joint function and alleviate inflammation and pain.

## 1. Introduction

Hyaluronic acid (HA) plays an important role in a wide range of medical physiological and pathological conditions: Notably, it finds application in dermatology, ophthalmology, cosmetic medicine, and rheumatology [1,2,3]. Its significance extends to wound healing, granulation, and cell migration [4]. However, the efficacy of HA in rheumatology remains a subject of controversy at times [5,6].

**Figure 1:**
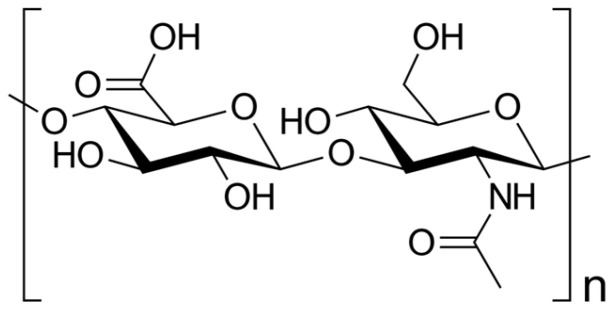
Skeletal formula of hyaluronan—a polymer consisting of D-glucuronic acid and *N*-acetyl-D-glucosamine linked via alternating β-(1→4) and β-(1→3) glycosidic bonds [7].

HA is as naturally occurring glycosaminoglycan composed of repeating disaccharide units consisting of glucuronic acids and N-acetylglucosamine (Figure 1), resulting in different molecular weights [7,8,9]. This structural variability imparts diverse functional implications in both physiological and pathological contexts [10]. HA is commercially produced through extraction from animal tissues, such as chicken combs, and from Streptococci bacteria [11].

Functionally, HA demonstrates remarkable water-binding capacity, rendering it an essential constituent of the extracellular matrix (ECM) [12]. Its elongated, unbranched chains create a gel-like network, imparting hydration and lubrication to crucial tissues like the skin, cartilage, and synovial fluid [13,14,15].

In the field of rheumatology, HA has garnered substantial attention, owing to its pivotal involvement in joint health and its relevance to diseases like osteoarthritis (OA) and rheumatoid arthritis (RA) [16,17].

OA is a degenerative joint disorder characterized by the gradual deterioration of articular cartilage, resulting in pain, stiffness, and diminished joint function [18]. There is no cure for OA, so doctors usually treat OA symptoms with a combination of therapies [18]. HA serves as a lubricant and shock absorber within the synovial fluid, facilitating smooth joint movements [19]. However, in OA, the concentration and quality of HA decrease, compromising its protective and viscoelastic properties due to heightened degradation and decreased synthesis. Consequently, this leads to impaired cartilage function and joint degeneration [20]. As a therapeutic approach, supplementation with exogenous HA has emerged to alleviate symptoms and enhance joint function in OA patients [21]. By restoring synovial fluid viscosity and promoting cartilage repair, HA aids in improving joint mobility and reducing pain [16].

On the other hand, RA is an autoimmune and inflammatory disease characterized by the immune system mistakenly attacking healthy cells, resulting in inflammation, particularly in the joints, leading to painful swelling [22]. RA can be effectively treated and managed with medication(s) and self-management strategies [22]. In RA, the level of HA in the synovial fluid is significantly diminished, causing reduced lubrication and increased inflammation and pain [23].

Numerous studies have explored the potential therapeutic benefits of exogenous HA supplementation in rheumatic diseases. Through intra-articular injections, HA has shown promise in improving joint mobility, reducing pain, and promoting cartilage repair by restoring synovial fluid viscosity [24].

Furthermore, HA has demonstrated immunomodulatory effects, including the suppression of pro-inflammatory cytokines and the promotion of anti-inflammatory cytokine production [25]. This suggests that HA may also hold promise in the treatment of immune-mediated rheumatic diseases [26].

Moreover, researchers have explored HA’s potential applications in drug delivery systems and tissue engineering due to its biocompatibility and biodegradability [27].

However, the use of HA therapy in rheumatology remains a topic of controversy, with conflicting evidence regarding its efficacy and safety [5,28].

In summary, HA represents a promising therapeutic option in the field of rheumatology due to its potential to enhance joint function and alleviate inflammation and pain [29]. Nevertheless, further investigation is required to fully elucidate its therapeutic potential in rheumatic diseases.

This review aims to provide a comprehensive overview of the structure, function, and rheumatological significance of HA. Additionally, it will discuss recent advancements in understanding HA’s role in joint health and the therapeutic potential of HA-based interventions. Furthermore, a critical analysis of the existing literature on HA in rheumatology will be presented.

## 2. Hyaluronic Acid: Structure, Function, and Biochemistry

HA plays a crucial role in diverse cellular and tissue processes, encompassing hydration, lubrication, tissue repair, regulation of inflammation, and cell signaling [14]. It is naturally synthesized by various cell types, predominantly fibroblasts, chondrocytes, and synoviocytes [30]. The biosynthesis of HA takes place in the plasma membrane through the coordinated activity of specific enzymes, including hyaluronan synthases [30].

### 2.1. Molecular Structure of Hyaluronic Acid

Hyaluronan synthases catalyze the addition of glucuronic acid and N-acetylglucosamine, leading to the formation of the repeating disaccharide units that constitute HA [31]. The molecular weight of HA displays highly variability, ranging from several hundred kilo Da to millions of kilo Da, exerting a direct impact on its functional properties [32]: Notably, higher-molecular-weight HA exhibits increased viscosity [33], thereby affecting its flow and lubrication capability, i.e., in joints [34]. As a result, high-molecular-weight HA provides superior lubrication and cushioning effects [35].

### 2.2. Biosynthesis and Degradation of Hyaluronic Acid

HA turnover in tissues is intricately regulated by a delicate balance between biosynthesis and degradation processes [36]. The degradation of HA primarily involves the action of enzymes known as hyaluronidases, which cleave HA into smaller fragments [37]. Hyaluronidase enzymes are categorized into several families, including HYAL1, HYAL2, and PH-20 [38], and they play a pivotal role in maintaining the appropriate concentration and size distribution of HA within tissues [39]. Furthermore, the degradation of HA can be modulated by reactive oxygen species, matrix metalloproteinases (MMPs), and other factors present in the extracellular environment [40].

### 2.3. Physiological Functions and Distribution in Tissues of Hyaluronic Acid

HA plays a critical role in tissue repair and remodeling processes within the human body [41]. It participates in various stages of wound healing, encompassing inflammation, cell migration, proliferation, and ECM remodeling [42]. As a scaffolding molecule, HA provides essential structural support and aids in cell migration during tissue repair [43] (Table 1).

During the inflammatory phase of wound healing, HA is involved in the recruitment and activation of immune cells, such as macrophages and neutrophils [51]. HA fragments generated during tissue injury can function as damage-associated molecular patterns and trigger immune responses [51]. Moreover, HA promotes the infiltration of immune cells into the wound site, facilitating the removal of debris and pathogens [52]. In the subsequent proliferative phase, HA contributes to cell migration and proliferation [53]. It forms a provisional matrix that guides cell movement and stimulates cell proliferation [53]. HA receptors, such as CD44 and RHAMM, mediate cell adhesion, migration, and signal transduction, enabling cells to migrate into the wound area and contribute to tissue repair [54]. Furthermore, HA plays a regulatory role in ECM remodeling during tissue repair [55]. It interacts with other components, such as fibronectin and collagen, promoting their assembly and organization [56]. HA also influences the activity of enzymes involved in ECM remodeling, such as MMPs and tissue inhibitors of MMPs, which are essential for matrix turnover and remodeling [57].

Beyond its involvement in tissue repair, HA also plays a role in tissue remodeling processes, such as embryonic development, organ morphogenesis, and angiogenesis [58]. HA provides a structural framework for cell migration and tissue organization during these processes [59]. It regulates cell behavior, including cell differentiation, proliferation, and survival, through interactions with specific receptors and signaling pathways [60].

Overall, HA exhibits a multifaceted role in tissue repair and remodeling, contributing to inflammation resolution, cell migration, proliferation, and ECM (re)organization [61]. Its involvement in these processes highlights its significance in wound healing, tissue regeneration, and developmental biology [62].

Regarding tissue distribution, HA is widely distributed throughout the body, with particularly high concentrations found in connective tissues, such as the skin, synovial fluid, and cartilage [63]. In the skin, HA resides in the ECM and contributes to tissue hydration, elasticity, and wound healing [64]. In the synovial fluid of joints, HA forms a viscous, gel-like substance that provides lubrication, shock absorption, and nutrient supply to the articular cartilage [65]. Additionally, HA is present in the vitreous humor of the eye, where it helps maintain the transparency and shape of the eyeball [66]. Moreover, HA is found in other tissues, including the umbilical cord, umbilical vessels, and embryonic tissues, where it plays crucial roles in development and tissue morphogenesis [67].

## 3. Pathophysiological Role of Hyaluronic Acid in Rheumatic Diseases

HA plays a crucial pathophysiological role in rheumatic diseases, especially concerning joint health and function [68]. In a healthy joint, HA’s viscoelastic properties facilitate smooth movement of the joint surfaces and shield the cartilage from excessive mechanical stress [69]. HA serves as a shock absorber, mitigating the impact on the joint and thereby reducing the risk of damage. However, in rheumatic conditions such as OA and RA, significant alterations occur in the HA metabolism and distribution within the joint [70].

### 3.1. Osteoarthritis

#### 3.1.1. Role of Hyaluronic Acid in Joint Lubrication

In OA, there is a notable reduction in the concentration of HA within the synovial fluid, leading to diminished joint lubrication and compromised cartilage protection [69]. This decline in HA levels can be attributed to an imbalance between HA synthesis and degradation, with increased activity of hyaluronidases, the enzymes responsible for HA breakdown [71]. Consequently, the cartilage becomes vulnerable to wear and tear, resulting in pain, stiffness, and functional impairment [72]. Dysregulated HA metabolism, including increased synthesis and degradation, contributes to cartilage degradation, synovitis, and pain in OA [73]. The altered biomechanical properties of HA affect joint lubrication, chondrocyte activity, and inflammation, further exacerbating disease progression [74].

#### 3.1.2. Protective Effects on Chondrocytes and Cartilage Matrix

HA serves as a fundamental component of the cartilage matrix, playing a crucial role in maintaining joint integrity. However, in OA, changes occur in the quantity, molecular weight, and distribution of HA, which can impact its functional properties [75]. The molecular weight of HA influences its retention time within the tissues [76]. Generally, higher-molecular-weight HA exhibits a longer half-life, meaning it remains in the joint or tissue for a more extended period before being broken down and cleared by the body [8]. This prolonged presence may result in longer-lasting effects [8]. On the other hand, lower-molecular-weight HA can more easily penetrate the ECM and reach target cells, while higher-molecular-weight HA may have more limited diffusion [8].

#### 3.1.3. Modulation of Inflammation and Synovial Fluid Changes

Inflammation is a hallmark of rheumatic diseases [77], and HA plays a multifaceted role in this process [78]. Pathological conditions often lead to changes in the quantity and molecular weight of HA in synovial fluid, with implications for disease severity and progression [79]. During the inflammatory phase, HA actively participates in the recruitment and activation of immune cells, such as macrophages and neutrophils [79]. HA engages with specific cell surface receptors, such as CD44 [80], and the binding affinity of HA to these receptors can vary depending on its molecular weight [81]. Distinct fractions of HA, characterized by different molecular weights, can have specific effects on cell signaling, inflammation, and tissue repair processes [82].

### 3.2. Rheumatoid Arthritis

In RA, the inflammatory process significantly disrupts HA homeostasis [83]. Synovial inflammation induces the release of pro-inflammatory cytokines and enzymes that promote the degradation of HA, leading to a decrease in its concentration and alterations in its molecular weight distribution [84]. These changes in HA metabolism have profound effects on joint lubrication, exacerbate cartilage damage, and perpetuate the inflammatory cycle [85].

#### 3.2.1. Altered Hyaluronic Acid Synthesis and Breakdown

In RA, alterations in HA synthesis and breakdown contribute to the pathogenesis of the disease [70]. Synovial fibroblasts, key players in RA pathophysiology, exhibit dysregulated HA synthesis, leading to increased production and accumulation of HA in the synovial fluid and tissues [86]. This abnormal HA synthesis is influenced by various factors, including pro-inflammatory cytokines and growth factors, which stimulate the expression of HA synthases [87]. Concurrently, increased HA degradation occurs due to upregulated expression and activity of hyaluronidases [88]. The imbalance between HA synthesis and degradation results in the accumulation of fragmented HA in the synovial fluid, exacerbating inflammation and joint damage [89]. Moreover, the presence of HA fragments in the synovium further amplifies the inflammatory response by activating immune cells and promoting the production of pro-inflammatory mediators [90]. The presence of HA fragments and their interaction with CD44 receptors contribute to the perpetuation of chronic inflammation and joint damage in RA [91].

#### 3.2.2. Inflammatory Mediator Modulation

Moreover, the anti-inflammatory properties of HA, which typically involve the modulation of the inflammatory responses within the joints, are disturbed in RA [83]. The inhibitory action of HA on the production of pro-inflammatory cytokines, such as tumor necrosis factor-alpha and interleukin-1 beta, and the suppression of inflammatory enzymes, such as cyclooxygenase-2 and MMPs, become dysregulated [92]. During the subsequent proliferative phase, HA continues to play a role in cell migration and proliferation, forming a provisional matrix that guides cell movement and stimulates cell proliferation [93].

#### 3.2.3. Effects on Synovial Hyperplasia and Pannus Formation

HA exerts significant effects on synovial hyperplasia and pannus formation in RA [93]. It plays a pivotal role in promoting synovial hyperplasia by stimulating the proliferation and migration of synovial fibroblasts [94]. Furthermore, it interacts with CD44 receptors on the surface of these fibroblasts, triggering intracellular signaling cascades that promote cell survival, proliferation, and ECM production [95]. Additionally, HA enhances the expression of various pro-inflammatory mediators, such as cytokines and chemokines, further driving synovial hyperplasia and inflammation [96]. Additionally, HA fragments, generated due to the increased breakdown of HA in RA, can stimulate the production of matrix-degrading enzymes, leading to cartilage and bone destruction [97].

## 4. Mechanisms of Action and Delivery Systems

HA has undergone extensive study to unravel its mechanisms of action and explore various delivery systems to enhance its therapeutic applications [29]. HA exerts its effects through multiple mechanisms, including its ability to bind to cell surface receptors, regulate cell signaling pathways, modulate inflammation, and provide structural support to tissues [98]. Moreover, HA serves as a viscoelastic agent, offering lubrication and shock absorption in joints, and contributing to tissue hydration and elasticity [99]. Overall, HA exhibits diverse mechanisms of action, and its therapeutic efficacy has been supported by clinical trials in various medical fields [100,101]. HA-based delivery systems have been developed to improve its delivery and enhance therapeutic outcomes [102]. As a relatively new polymer for constructing drug delivery systems, HA offers a promising platform for physically encapsulating or chemically conjugating various drugs [103]. With a favorable safety profile, HA-based therapies have shown promise as effective treatments for conditions such as OA, among others [104]. It is essential to note that the specific effects of HA, based on its molecular weight, can vary depending on the context, such as the route of administration (e.g., intra-articular injection, topical application) and the specific medical condition being targeted [105]. The selection of HA with a specific molecular weight for therapeutic use is often based on the desired therapeutic outcomes and the specific needs of the patient [106].

### 4.1. Safety Profile and Adverse Events

In terms of safety, HA-based therapies are generally well-tolerated [107]. It is crucial for healthcare professionals to conduct a thorough evaluation of patients for contraindications, carefully select suitable candidates for HA-based therapies, and adhere to proper injection techniques to minimize the risk of adverse events. Furthermore, patients should be informed about potential risks and benefits, and their consent should be obtained before initiating treatment.

While HA is considered safe, it is essential to be aware of potential local and systemic reactions, the risk of infection, hypersensitivity reactions, and long-term safety when using HA-based therapies [108]. HA derived from different sources and with varying molecular weights may differ in immunogenic potential [109]. Some studies have indicated that lower-molecular-weight HA might have a higher likelihood of inducing immune responses [110], although the clinical significance of this is still under investigation. Adherence to appropriate guidelines, proper patient selection, and adequate follow-up(s) are essential aspects to ensure the safe and effective use of HA in clinical practice.

#### 4.1.1. Local and Systemic Reactions

Adverse events related to HA-based therapies are generally rare and mostly mild, often limited to local reactions at the injection site, such as pain or swelling [111]. Severe allergic reactions or systemic adverse events are extremely uncommon but can occur, underscoring the importance of proper patient selection and administration technique [112]. Overall, the safety profile of HA is considered favorable (Table 2).

#### 4.1.2. Infections Risks

In terms of infection risk, the incidence of HA-related infections is low [108]. Adherence to proper aseptic techniques during the procedure and following guidelines can effectively minimize the risk of infection [113]. However, in rare cases, infection can occur, particularly when HA injections are performed in areas with compromised skin integrity or in the presence of preexisting infections [108].

#### 4.1.3. Hypersensitivity Reactions

Hypersensitivity reactions to HA have been reported, albeit rarely [1]. These reactions can vary from mild local allergic reactions to more severe systemic manifestations [114]. Individuals with a history of known hypersensitivity to HA or other components of the formulation should be carefully evaluated and, if necessary, alternative treatment options should be considered.

#### 4.1.4. Long-Term Safety

Long-term safety and follow-up studies play a crucial role in assessing the safety profile of HA-based therapies. Although HA has been utilized for several decades, ongoing research aims to evaluate its long-term effects and ensure its continued safety. Follow-up studies have consistently demonstrated the safety and efficacy of HA injections over extended periods, with low rates of serious adverse events reported [24] (Table 2).

**Table 2:** Safety and adverse events of hyaluronic acid (HA) in rheumatology.

Study	Design	Participants	Findings	References
Petrella et al. (2008)	Systematic review	24 randomized controlled trials	HA injections had a low incidence of adverse events, with most being mild and transient in nature.	[111]
Shen et al. (2018)	Meta-analysis	84 randomized controlled trials	HA injections were generally well tolerated, with low rates of serious adverse events.	[115]
Najm et al. (2021)	Systematic review and meta-analysis	15 randomized controlled trials	HA injections demonstrated a favourable safety profile, with rare reports of hypersensitivity reactions and infections.	[116]
Conrozier et al. (2021)	Review (Delphi method)	24 statements	HA injections were associated with a low risk of adverse events, with most being localized and self-limiting.	[117]
Chevalier et al. (2020)	Systematic review and meta-analysis	162 randomized controlled trials	HA injections were well tolerated, with a low incidence of serious adverse events and local reactions.	[118]

Summary of selected studies that have examined the safety profile and adverse events associated with the use of HA in rheumatology. It is important to note that the incidence and nature of adverse events may vary across studies and patient populations. For a comprehensive understanding, it is recommended to refer to the full-text articles cited in the references.

### 4.2. Systemic Administration of Hyaluronic Acid

To optimize the delivery of HA-based therapeutics, various delivery systems have been developed. These systems encompass injectable gels, nanoparticles, liposomes, and hydrogels, which enable the controlled release and targeted delivery of HA to specific tissues or cells [119]. By utilizing these delivery systems, the bioavailability and retention of HA at the desired site can be enhanced, thereby improving its therapeutic efficacy [120].

#### 4.2.1. Oral, Intravenous, and Intraarticular Routes

HA has been extensively studied for its potential therapeutic applications via different routes of administration, including oral, intra-articular, and intravenous routes [121]. Each route presents its own set of challenges and limitations.

The oral administration of HA aims to provide systemic benefits, targeting various tissues throughout the body [122]. However, the oral bioavailability of HA is generally low, due to its large molecular size and susceptibility to enzymatic degradation in the gastrointestinal tract [123]. Furthermore, the absorption of HA through the intestinal epithelium is limited [124]. These challenges have hindered the development of effective oral formulations of HA, and alternative routes of administration have been explored to overcome these limitations.

Intra-articular administration of HA has been widely used for the treatment of joint disorders such as OA [16]. This route allows for the direct delivery of HA into the affected joint, providing local therapeutic effects. Intra-articular injections of HA have shown clinical efficacy in reducing pain, improving joint function, and delaying disease progression [125] (Table 3). However, the limitations of this route include the need for repeated injections, potential injection-related complications, and the possibility of adverse reactions, although these are rare [16].

Intravenous administration of HA has been investigated for its potential systemic effects and applications. This route allows the rapid distribution of HA throughout the body, potentially targeting various tissues and organs. However, the challenges lie in achieving optimal bioavailability and targeting specific tissues, as HA may undergo rapid clearance from the bloodstream and may not reach the desired site of action in sufficient concentrations [130].

#### 4.2.2. Delivery Systems

In summary, due to its unique physicochemical properties and therapeutic potential, HA has garnered considerable attention in terms of therapeutical use. However, the clinical utility of HA is hindered by its short half-life and poor bioavailability [131]. To overcome these limitations, various delivery systems have been developed to enhance the stability, sustained release, and targeted delivery of HA [29,104]. Different types of HA delivery systems have been explored, such as injectable hydrogels, including micro- and nanoparticles, liposomes, as well as coatings and scaffolds [132,133,134,135]. Furthermore, various formulation strategies and modes of action, such as crosslinking techniques, surface modification, encapsulation methods, or sustained release mechanisms, have been employed [136,137,138,139]. Moreover, hybrid delivery systems have been introduced, allowing for optimized HA delivery, and contributing to personalized treatment options [140,141].

### 4.3. Disease-Modifying Effects and Immunomodulation

HA appears to have the ability to modify the disease course and modulate the immune response in rheumatological conditions [142,143]. This is attributed to its interactions with receptors, impact on inflammatory mediators, and modulation of cellular responses [144] (see also Section 2 and Section 3 above: Physiological and pathophysiological role of HA). Understanding of HA’s therapeutic potential in rheumatologic disorders paves the way for the development of novel treatment strategies.

### 4.4. Challenges and Limitations

Despite the therapeutic potential of HA, its clinical application via different routes still faces certain limitations. These include the high cost of production and purification, potential immunogenicity, and allergic reactions, as well as the need for further optimization of delivery systems to enhance bioavailability, targeting, and sustained release [145]. Additionally, there is a need for standardized dosing regimens, well-designed clinical trials, and long-term safety data to establish the efficacy and safety profile of HA-based therapies.

In conclusion, while HA holds promise for therapeutic applications via oral, intra-articular, and intravenous routes, each route presents specific challenges and limitations. Further research is needed to overcome these limitations, optimize delivery systems, and establish the efficacy, safety, and long-term benefits of HA-based therapies.

## 5. Therapeutic Approaches

Understanding the pathophysiological role of HA in rheumatic diseases offers potential avenues for therapeutic interventions. Clinical trials have been conducted to evaluate the efficacy of HA-based therapies in various medical conditions. In the field of rheumatology, intra-articular injections of HA have shown beneficial effects in reducing pain, improving joint function, and delaying the progression of OA [46]. Clinical evidence supports the use of HA injections as a safe and effective treatment option for knee OA, with long-term benefits and a favorable safety profile [146].

Targeting HA-mediated pathways, including HA synthesis, degradation, and interactions with specific receptors, holds promise for developing disease-modifying therapies [147]. Additionally, emerging techniques like tissue engineering and nanotechnology present exciting opportunities for HA-based interventions [148].

### 5.1. Combination Therapies

HA has demonstrated potential for use in combination therapies and novel approaches to enhance its therapeutic effects in various medical conditions [149]. These approaches involve combinations of HA with corticosteroids, non-steroidal anti-inflammatory drugs (NSAIDs), or platelet-rich plasma (PRP), which can provide synergistic benefits in managing inflammatory and degenerative conditions.

#### 5.1.1. Hyaluronic Acid and Corticosteroids

The combination of HA and corticosteroids has been investigated in joint disorders such as OA, demonstrating improved pain relief and functional outcomes compared to individual treatments [150,151].

#### 5.1.2. Hyaluronic Acid and Nonsteroidal Anti-Inflammatory Drugs

Another combination strategy involves the use of HA in conjunction with NSAIDs. This approach aims to address both the inflammatory component and symptomatic relief in conditions such as OA [152]. Studies have shown that combining HA with NSAIDs can result in enhanced pain reduction and improved joint function compared to NSAIDs alone [153].

#### 5.1.3. Hyaluronic Acid and Platelet-Rich Plasma

Furthermore, HA has been explored in combination with PRP, which contains growth factors and cytokines that promote tissue healing and regeneration [154]. The combination of HA and PRP has shown promising results in accelerating tissue repair, reducing pain, and improving functional outcomes in conditions such as tendinopathies and OA [155].

### 5.2. Novel Approaches, Emerging Strategies, and Future Directions

HA plays a key role in the pathophysiology of rheumatic diseases, exerting diverse effects on inflammation, joint destruction, and tissue homeostasis [156]. Advances in understanding HA metabolism and its interaction with immune and non-immune components have illuminated the complex mechanisms underlying rheumatic diseases. Leveraging this knowledge may lead to the development of innovative therapeutic strategies to improve patient outcomes and alleviate the burden associated with rheumatic diseases.

Novel approaches are also being explored to enhance the therapeutic potential of HA. These include the development of modified forms of HA with improved properties, such as crosslinked HA derivatives, which exhibit the prolonged residence time and sustained release of HA [157]. Additionally, nanotechnology-based delivery systems and combinations with other biomaterials are being investigated to improve HA’s bioavailability, targeting, and regenerative potential [158].

Emerging strategies for HA-based therapies involve tissue engineering approaches where HA is combined with cells and scaffolds to promote tissue regeneration [159]. This approach holds promise for the repair and regeneration of various tissues, including cartilage, skin, and bone [160].

By harnessing the synergistic effects of HA with other therapeutic agents, it is possible to enhance its regenerative, anti-inflammatory, and analgesic properties, opening up new avenues for personalized medicine and tissue engineering approaches [161,162,163]. Further research is warranted to elucidate the precise molecular mechanisms and evaluate the efficacy of targeted interventions in clinical settings with respect to long-term safety and cost-effectiveness of combination therapies involving HA.

## 6. Conclusions and Future Perspectives

In conclusion, HA has emerged as a valuable therapeutic agent in rheumatology due to its unique properties, including its lubricating, anti-inflammatory, and chondroprotective effects, making it an attractive option for the management of various rheumatic diseases, particularly OA. Intra-articular injections of HA have shown efficacy in reducing pain, improving joint function, and delaying disease progression. Moreover, HA’s role in tissue repair and remodeling, as well as its potential to modulate inflammation and protect chondrocytes and cartilage matrices, further highlight its therapeutic potential in rheumatology.

In clinical practice, intra-articular injections of exogenous HA have been widely used as a therapeutic approach for managing rheumatic diseases. These injections aim to restore depleted levels of HA in the joint, improve lubrication, reduce inflammation, and provide symptomatic relief. Numerous clinical studies have demonstrated the efficacy of HA injections in alleviating pain, enhancing joint function, and delaying the need for surgical interventions in patients with OA and RA.

Insurance coverage for medical procedures, including intra-articular HA injections, can vary widely depending on the country, specific insurance plan, and local regulations. This may be due to a lack of sufficient evidence. Health insurance systems often consider the effectiveness and cost-effectiveness of treatments before providing coverage. If there is limited scientific evidence or conflicting studies regarding the effectiveness of intra-articular HA injections for a specific condition, insurers may be hesitant to cover it. Intra-articular HA injections can be relatively expensive, especially considering the number of injections required for a full treatment course. If insurers deem the cost to outweigh the potential benefits, they may choose not to cover it. Additionally, in some cases, there may be alternative treatments available for the same condition that might have been proven to be more effective or cost-effective. Then, insurers may prioritize coverage for those alternatives, such as physical therapy, medications, or other interventions, over intra-articular HA injections.

Future perspectives on the use of HA in rheumatology are promising. Advancements in the development of delivery systems and formulations may improve the bioavailability and sustained release of HA, thereby optimizing its therapeutic effects. Combination therapies involving HA, such as its combination with corticosteroids, NSAIDs, or PRP, hold potential for enhanced therapeutic outcomes and improved patient management. Furthermore, the exploration of novel approaches, including tissue engineering and regenerative medicine strategies, may lead to the development of more targeted and personalized treatment options in rheumatic diseases.

Despite the progress made in understanding the role of HA in rheumatology, further research is needed to elucidate its mechanisms of action, optimize treatment protocols, and establish long-term safety and efficacy. Well-designed clinical trials, standardized dosing regimens, and comprehensive follow-up studies are necessary to gather robust evidence and guide clinical practice. Additionally, cost-effectiveness analyses and health–economic evaluations will contribute to the broader adoption and accessibility of HA-based therapies in rheumatology.

Overall, HA holds great promise as a therapeutic agent in rheumatology. With ongoing research and technological advancements, it is expected that HA-based treatments will continue to evolve, providing improved outcomes and enhancing the quality of life for patients with rheumatic diseases.

## Figures and Tables

**Table 1:** Physiological functions of hyaluronic acid (HA) in rheumatology.

Function	Mechanisms	References
Lubrication of joints	HA provides lubrication and viscoelastic properties to synovial fluid, reducing friction between joint surfaces and enhancing joint mobility.	[44]
Chondroprotection	HA exhibits chondroprotective effects by promoting cartilage matrix synthesis, reducing matrix degradation, and inhibiting the activity of proteolytic enzymes	[45,46]
Anti-inflammatory activity	HA can modulate inflammation by reducing the expression of pro-inflammatory cytokines and enzymes, inhibiting leukocyte migration, and suppressing immune responses.	[47,48]
Tissue repair and remodeling	HA plays a role in tissue repair and remodeling processes by promoting cell migration, angiogenesis, and extracellular matrix remodeling	[44,49]
Viscoelasticity	HA contributes to the viscoelastic properties of connective tissues, maintaining tissue integrity, elasticity, and shock-absorbing capabilities.	[48,50]

Selected studies on the physiological role of HA in rheumatology. This is not an exhaustive list, and further research and clinical trials have been conducted in this field. For more detailed information, it is recommended to refer to the referenced papers.

**Table 3:** Intra-articular application of hyaluronic acid (HA) in osteoarthritis (OA) and its findings.

Study	Design	Participants	Intervention	Findings	References
Altman et al. (2004)	Randomized, double-blind, placebo-controlled trial	495 patients with knee OA	HA injections (3 weekly injections) vs. placebo	HA group showed significant improvement in pain and function compared to placebo	[126]
Bannuru et al. (2009)	Meta-analysis	29 randomized controlled trials	HA injections vs. control interventions (placebo, saline, or NSAIDs)	HA injections were superior to control interventions in reducing pain and improving function in knee OA	[127]
Rutjes et al. (2012)	Systematic review and meta-analysis	76 randomized controlled trials	HA injections vs. control interventions (placebo or no treatment)	HA injections provided significant pain relief and functional improvement compared to control interventions in knee OA	[128]
Filardo et al. (2015)	Randomized controlled trial	160 patients with knee OA	HA injections vs. platelet-rich plasma (PRP) injections	HA and PRP injections showed similar efficacy in reducing pain and improving function in knee osteoarthritis	[45]
Bannuru et al. (2015)	Systematic review and meta-analysis	137 randomized controlled trials	Various HA preparations vs. control interventions (placebo, saline, or corticosteroids)	HA injections were effective in reducing pain and improving function in knee OA with a favourable safety profile	[129]

Selected clinical studies on the use of intraarticular HA injections in OA. This is not an exhaustive list, and further research and clinical trials have been conducted in this field. For more detailed information, it is recommended to refer to the referenced papers.

## Data Availability

No new data were created or analyzed in this review article. Data sharing is not applicable to this review.
